# Methyl 4-(4-bromo­anilino)-2′,5-dioxo-5*H*-spiro­[furan-2,3′-indoline]-3-carboxyl­ate

**DOI:** 10.1107/S1600536814001329

**Published:** 2014-01-29

**Authors:** Rajeswari Gangadharan, Selvarangam E. Kiruthika, K. Sethusankar, P. T. Perumal

**Affiliations:** a Department of Physics, Ethiraj College for Women (Autonomous), Chennai 600 008, India; b Organic Chemistry Division, Central Leather Research Institute, Adyar, Chennai 600 020, India; c Department of Physics, RKM Vivekananda College (Autonomous), Chennai 600 004, India

## Abstract

In the title compound, C_19_H_13_BrN_2_O_5_, the spiro furan ring is almost planar with a maximum deviation of 0.034 (2) Å. The indole unit and the furan ring are normal to each other, making a dihedral angle of 87.82 (8) °. The mol­ecular structure is stabilized by an intra­molecular N—H⋯O hydrogen bond, which generates an *S*(6) ring motif. In the crystal, mol­ecules are linked *via* pairs of N—H⋯O hydrogen bonds, forming inversion dimers enclosing *R* ^2^_2_(8) ring motifs.

## Related literature   

For applications of oxindoles, see: Akai *et al.* (2004 ▶); Gallagher *et al.* (1985 ▶); Tokunaga *et al.* (2001 ▶); Zaveri *et al.* (2004 ▶). For applications of tetra­hydro­furans, see: Garzino *et al.* (2000 ▶). For a related structure, see: Gangadharan *et al.* (2013 ▶). For the length of a C—Br single bond, see: Koşar *et al.* (2006 ▶). For resonance structure in a carboxyl­ate group, see: Merlino (1971 ▶); Varghese *et al.* (1986 ▶). For graph-set notation, see: Bernstein *et al.* (1995 ▶).
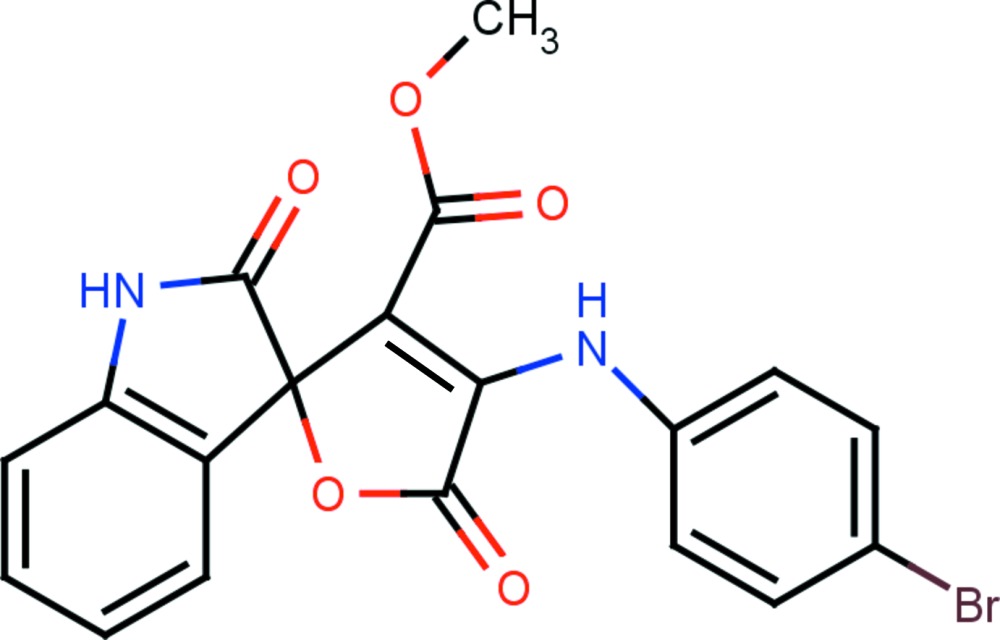


## Experimental   

### 

#### Crystal data  

C_19_H_13_BrN_2_O_5_
*M* *_r_* = 429.21
Triclinic, 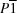

*a* = 7.845 (5) Å
*b* = 8.365 (5) Å
*c* = 13.703 (5) Å
α = 81.565 (5)°
β = 81.944 (5)°
γ = 76.183 (5)°
*V* = 858.6 (8) Å^3^
*Z* = 2
Mo *K*α radiation
μ = 2.43 mm^−1^
*T* = 296 K
0.30 × 0.25 × 0.20 mm

#### Data collection  

Bruker SMART APEXII area-detector diffractometer
Absorption correction: multi-scan (*SADABS*; Bruker, 2008 ▶) *T*_min_ = 0.487, *T*_max_ = 0.615
19580 measured reflections
5243 independent reflections
3611 reflections with *I* > 2σ(*I*)
*R*_int_ = 0.032

#### Refinement  

*R*[*F* ^2^ > 2σ(*F* ^2^)] = 0.034
*wR*(*F* ^2^) = 0.095
*S* = 1.02
5243 reflections
251 parameters
H atoms treated by a mixture of independent and constrained refinement
Δρ_max_ = 0.30 e Å^−3^
Δρ_min_ = −0.31 e Å^−3^

### 

Data collection: *APEX2* (Bruker, 2008 ▶); cell refinement: *SAINT* (Bruker, 2008 ▶); data reduction: *SAINT*; program(s) used to solve structure: *SHELXS97* (Sheldrick, 2008 ▶); program(s) used to refine structure: *SHELXL97* (Sheldrick, 2008 ▶); molecular graphics: *ORTEP-3 for Windows* (Farrugia, 2012 ▶); software used to prepare material for publication: *SHELXL97* and *PLATON* (Spek, 2009 ▶).

## Supplementary Material

Crystal structure: contains datablock(s) global, I. DOI: 10.1107/S1600536814001329/su2688sup1.cif

Structure factors: contains datablock(s) I. DOI: 10.1107/S1600536814001329/su2688Isup2.hkl

Supporting information file. DOI: 10.1107/S1600536814001329/su2688Isup3.cml

CCDC reference:

Additional supporting information:  crystallographic information; 3D view; checkCIF report

## Figures and Tables

**Table 1:** Hydrogen-bond geometry (Å, °)

*D*—H⋯*A*	*D*—H	H⋯*A*	*D*⋯*A*	*D*—H⋯*A*
N2—H2*A*⋯O5	0.84 (2)	2.06 (3)	2.773 (3)	143 (2)
N1—H1*A*⋯O1^i^	0.92 (2)	1.96 (2)	2.869 (3)	168 (2)

Symmetry code: (i) 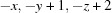.

**Fig. 1.**
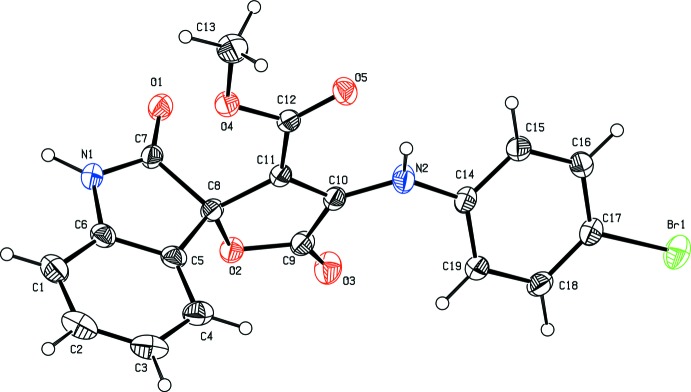
The molecular structure of the title compound, with atom labelling. Displacement ellipsoids are drawn at 30% probability level.

**Fig. 2.**
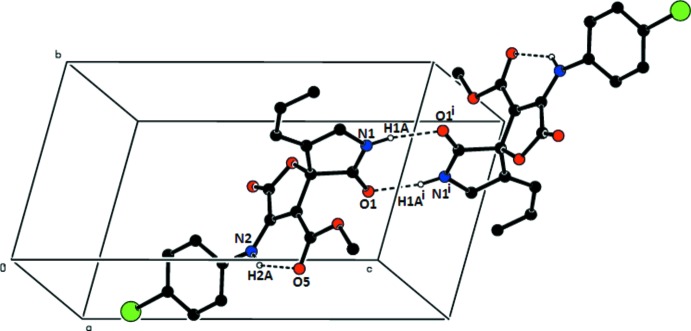
Part of crystal packing of the title compound, showing the formation of the intramolecular S(6) ring motif and the R22(8)inversion dimer, as viewed along the b-axis [see Table 1 for details of the hydrogen bonding (dashed lines); symmetry code: (i) -x, - y + 1, - z + 2].

## supplementary crystallographic information

### 1. Comment

The indole template is generally recognized as an important structure in medicinal chemistry. In particular oxindoles are important constituents of drugs (Akai *et al.*, 2004). The Oxindole motif is present in the anti-Parkinson's drug ropinirole (Gallagher *et al.*, 1985), in non-opioid receptor ligands (Zaveri *et al.*, 2004) and in growth hormone secretagogues (Tokunaga *et al.*, 2001). Tetrahydrofuran is a common motif which can be found in numerous natural products such as polyether antibiotics, nucleosides and lignans (Garzino *et al.*, 2000).

The five- (N1/C1–C8) and six- (C1–C6) membered rings in the indole unit are coplanar, making a dihedral angle of 1.50 (9)°. The indole moiety is orthogonal to the furan ring as indicated by the dihedral angle of 87.82 (8)°. The benzene ring is bisectionally oriented to the furan ring with a dihedral angle 37.54 (10)°. The bond lengths and angles are comparable with those in a similar structure (Gangadharan *et al.*, 2013). In addition, the C–Br bond distance of 1.896 (2) Å, is slightly shorter than the value reported for the C–Br single bond (1.961 (3) Å; Koşar *et al.*, 2006). The C15–C16–C17–Br1 torsion angle of 177.77 (14) ° indicates that the bromine atom is antiperiplanar to the benzene ring.

The keto O atoms O3 and O1 deviate from the furan and indoline rings by 0.130 (1) Å and 0.043 (1) Å, respectively. The sum of the bond angles around the nitrogen atoms N1 [359.9 (44) °] and N2 [360.0 (49) °] suggests *sp*^2^ hybridization. The significant difference in length of the C12–O4 = 1.338 (2) Å and C13–O4 = 1.442 (2) Å bonds is attributed to partial contribution from O^-^–C=O^+^–C resonance structure of the O3═ C12–O4–C13 group (Merlino, 1971). This feature is commonly observed in carboxyl ester groups of the substituents in various compounds where the average distances are 1.340 Å and 1.447 Å, respectively (Varghese *et al.*, 1986). The molecular structure is stabilized by an intramolecular N—H···O hydrogen bond which generates an S(6) ring motif (Table 1).

In the crystal, molecules are linked via pairs of N-H···O hydrogen bonds forming inversion dimers enclosing *R*^2^_2_(8) ring motifs (Bernstein *et al.*, 1995; Table 1 and Fig. 2).

### 2. Experimental

Isatin (1 mmol), *p*-bromoaniline (1 mmol), and dimethyl acetylene dicarboxylate (DMAD; 1 mmol) were stirred at room temperature in methanol in the presence of Triethylamine (20 mol %) for 4 hrs. The solid formed was filtered and recrystallized from methanol to afford the title compound as a pure yellow solid (85% yield).

### 3. Refinement

The hydrogen atoms were located in difference electron density maps. The H-atoms of the amine groups were refined with distance restraints of N—H = 0.89 (2) Å with *U*_iso_(H) = 1.2*U*_eq_(N). The C bound H atoms were included in calculated positions and treated as riding atoms: C—H = 0.93 and 0.96 Å for CH and CH_3_ H atoms, respectively, with *U*_iso_(H) = 1.5*U*_eq_(C-methyl) and = 1.2*U*_eq_(C) for other H atoms.

### Crystal data

**Table:**

C_19_H_13_BrN_2_O_5_	*Z* = 2
*M**_r_* = 429.21	*F*(000) = 432
Triclinic, *P*1	*D*_x_ = 1.660 Mg m^−^^3^
Hall symbol: -P 1	Mo *K*α radiation, λ = 0.71073 Å
*a* = 7.845 (5) Å	Cell parameters from 3611 reflections
*b* = 8.365 (5) Å	θ = 2.5–30.7°
*c* = 13.703 (5) Å	µ = 2.43 mm^−^^1^
α = 81.565 (5)°	*T* = 296 K
β = 81.944 (5)°	Block, yellow
γ = 76.183 (5)°	0.30 × 0.25 × 0.20 mm
*V* = 858.6 (8) Å^3^	

### Data collection

**Table:**

Bruker SMART APEXII area-detector diffractometer	5243 independent reflections
Radiation source: fine-focus sealed tube	3611 reflections with *I* > 2σ(*I*)
Graphite monochromator	*R*_int_ = 0.032
ω and φ scans	θ_max_ = 30.7°, θ_min_ = 2.5°
Absorption correction: multi-scan (*SADABS*; Bruker, 2008)	*h* = −11→11
*T*_min_ = 0.487, *T*_max_ = 0.615	*k* = −10→11
19580 measured reflections	*l* = −19→19

### Refinement

**Table:**

Refinement on *F*^2^	Primary atom site location: structure-invariant direct methods
Least-squares matrix: full	Secondary atom site location: difference Fourier map
*R*[*F*^2^ > 2σ(*F*^2^)] = 0.034	Hydrogen site location: inferred from neighbouring sites
*wR*(*F*^2^) = 0.095	H atoms treated by a mixture of independent and constrained refinement
*S* = 1.02	*w* = 1/[σ^2^(*F*_o_^2^) + (0.0509*P*)^2^ + 0.0269*P*] where *P* = (*F*_o_^2^ + 2*F*_c_^2^)/3
5243 reflections	(Δ/σ)_max_ = 0.001
251 parameters	Δρ_max_ = 0.30 e Å^−^^3^
0 restraints	Δρ_min_ = −0.31 e Å^−^^3^

### Special details

**Table:**

Geometry. All e.s.d.'s (except the e.s.d. in the dihedral angle between two l.s. planes) are estimated using the full covariance matrix. The cell e.s.d.'s are taken into account individually in the estimation of e.s.d.'s in distances, angles and torsion angles; correlations between e.s.d.'s in cell parameters are only used when they are defined by crystal symmetry. An approximate (isotropic) treatment of cell e.s.d.'s is used for estimating e.s.d.'s involving l.s. planes.
Refinement. Refinement of *F*^2^ against ALL reflections. The weighted *R*-factor *wR* and goodness of fit *S* are based on *F*^2^, conventional *R*-factors *R* are based on *F*, with *F* set to zero for negative *F*^2^. The threshold expression of *F*^2^ > σ(*F*^2^) is used only for calculating *R*-factors(gt) *etc*. and is not relevant to the choice of reflections for refinement. *R*-factors based on *F*^2^ are statistically about twice as large as those based on *F*, and *R*- factors based on ALL data will be even larger.

### Fractional atomic coordinates and isotropic or equivalent isotropic displacement parameters (Å^2^)

**Table:**

	*x*	*y*	*z*	*U*_iso_*/*U*_eq_	
C1	−0.4990 (3)	0.6472 (2)	0.90768 (17)	0.0504 (5)	
H1	−0.5215	0.6959	0.9664	0.060*	
C2	−0.6306 (3)	0.6597 (3)	0.8475 (2)	0.0582 (6)	
H2	−0.7443	0.7179	0.8665	0.070*	
C3	−0.5985 (3)	0.5890 (2)	0.76091 (18)	0.0558 (6)	
H3	−0.6902	0.5995	0.7225	0.067*	
C4	−0.4295 (3)	0.5013 (2)	0.72958 (16)	0.0466 (5)	
H4	−0.4066	0.4538	0.6705	0.056*	
C5	−0.2984 (2)	0.48741 (19)	0.78896 (13)	0.0364 (4)	
C6	−0.3335 (2)	0.5594 (2)	0.87638 (13)	0.0380 (4)	
C7	−0.0421 (2)	0.4317 (2)	0.87382 (12)	0.0337 (4)	
C8	−0.1067 (2)	0.40203 (19)	0.77657 (12)	0.0332 (3)	
C9	0.0787 (2)	0.3804 (2)	0.62970 (13)	0.0370 (4)	
C10	0.0397 (2)	0.2139 (2)	0.66609 (12)	0.0340 (4)	
C11	−0.0593 (2)	0.22549 (19)	0.75528 (12)	0.0329 (3)	
C12	−0.0965 (2)	0.0843 (2)	0.82184 (12)	0.0334 (3)	
C13	−0.2248 (3)	−0.0121 (2)	0.97751 (15)	0.0555 (5)	
H13A	−0.3023	−0.0637	0.9511	0.083*	
H13B	−0.2802	0.0271	1.0392	0.083*	
H13C	−0.1165	−0.0913	0.9885	0.083*	
C14	0.2003 (2)	0.0426 (2)	0.53099 (13)	0.0366 (4)	
C15	0.3175 (3)	−0.1104 (2)	0.52758 (14)	0.0429 (4)	
H15	0.3302	−0.1848	0.5850	0.052*	
C16	0.4145 (3)	−0.1526 (2)	0.44021 (14)	0.0424 (4)	
H16	0.4923	−0.2554	0.4381	0.051*	
C17	0.3960 (2)	−0.0425 (2)	0.35616 (13)	0.0389 (4)	
C18	0.2789 (3)	0.1086 (2)	0.35738 (13)	0.0426 (4)	
H18	0.2666	0.1819	0.2995	0.051*	
C19	0.1797 (3)	0.1509 (2)	0.44496 (13)	0.0418 (4)	
H19	0.0990	0.2523	0.4461	0.050*	
N1	−0.1789 (2)	0.52477 (18)	0.92420 (11)	0.0400 (3)	
N2	0.1045 (2)	0.07406 (19)	0.62338 (12)	0.0440 (4)	
O1	0.10713 (17)	0.37852 (16)	0.89603 (10)	0.0427 (3)	
O2	−0.01219 (16)	0.48646 (14)	0.69475 (9)	0.0378 (3)	
O3	0.17429 (19)	0.42333 (17)	0.56122 (10)	0.0513 (3)	
O4	−0.18764 (17)	0.12567 (14)	0.90801 (9)	0.0405 (3)	
O5	−0.04501 (18)	−0.05806 (15)	0.80338 (10)	0.0458 (3)	
Br1	0.53764 (3)	−0.10026 (3)	0.237127 (14)	0.05583 (10)	
H1A	−0.171 (3)	0.566 (3)	0.9820 (18)	0.067*	
H2A	0.077 (3)	−0.004 (3)	0.6630 (19)	0.067*	

### Atomic displacement parameters (Å^2^)

**Table:**

	*U*^11^	*U*^22^	*U*^33^	*U*^12^	*U*^13^	*U*^23^
C1	0.0486 (12)	0.0401 (10)	0.0558 (12)	−0.0017 (9)	0.0012 (10)	−0.0043 (9)
C2	0.0434 (12)	0.0434 (11)	0.0796 (17)	−0.0040 (9)	−0.0056 (11)	0.0091 (11)
C3	0.0466 (12)	0.0486 (11)	0.0721 (16)	−0.0154 (9)	−0.0230 (11)	0.0164 (11)
C4	0.0535 (12)	0.0409 (10)	0.0489 (11)	−0.0160 (9)	−0.0179 (9)	0.0048 (8)
C5	0.0427 (10)	0.0273 (8)	0.0390 (9)	−0.0086 (7)	−0.0089 (8)	0.0025 (7)
C6	0.0418 (10)	0.0294 (8)	0.0403 (9)	−0.0062 (7)	−0.0032 (8)	−0.0003 (7)
C7	0.0426 (10)	0.0303 (8)	0.0296 (8)	−0.0098 (7)	−0.0047 (7)	−0.0048 (6)
C8	0.0411 (9)	0.0319 (8)	0.0280 (8)	−0.0107 (7)	−0.0057 (7)	−0.0025 (6)
C9	0.0434 (10)	0.0384 (9)	0.0314 (8)	−0.0120 (8)	−0.0064 (7)	−0.0039 (7)
C10	0.0407 (9)	0.0334 (8)	0.0296 (8)	−0.0093 (7)	−0.0061 (7)	−0.0054 (6)
C11	0.0398 (9)	0.0312 (8)	0.0289 (8)	−0.0074 (7)	−0.0071 (7)	−0.0045 (6)
C12	0.0368 (9)	0.0335 (8)	0.0307 (8)	−0.0076 (7)	−0.0073 (7)	−0.0029 (6)
C13	0.0698 (14)	0.0489 (11)	0.0409 (11)	−0.0155 (10)	0.0080 (10)	0.0075 (9)
C14	0.0447 (10)	0.0385 (9)	0.0296 (8)	−0.0141 (8)	−0.0013 (7)	−0.0081 (7)
C15	0.0595 (12)	0.0342 (9)	0.0328 (9)	−0.0087 (8)	−0.0039 (8)	−0.0008 (7)
C16	0.0509 (11)	0.0354 (9)	0.0377 (10)	−0.0039 (8)	−0.0019 (8)	−0.0062 (7)
C17	0.0456 (10)	0.0425 (9)	0.0322 (9)	−0.0163 (8)	−0.0016 (7)	−0.0082 (7)
C18	0.0604 (12)	0.0399 (9)	0.0303 (9)	−0.0163 (9)	−0.0104 (8)	0.0000 (7)
C19	0.0538 (11)	0.0357 (9)	0.0360 (9)	−0.0053 (8)	−0.0120 (8)	−0.0060 (7)
N1	0.0453 (9)	0.0400 (8)	0.0351 (8)	−0.0048 (7)	−0.0057 (7)	−0.0123 (6)
N2	0.0628 (11)	0.0354 (8)	0.0331 (8)	−0.0137 (7)	0.0063 (7)	−0.0081 (6)
O1	0.0403 (7)	0.0487 (7)	0.0402 (7)	−0.0053 (6)	−0.0085 (6)	−0.0126 (6)
O2	0.0499 (7)	0.0329 (6)	0.0316 (6)	−0.0140 (5)	−0.0012 (5)	−0.0026 (5)
O3	0.0622 (9)	0.0502 (8)	0.0425 (8)	−0.0225 (7)	0.0098 (7)	−0.0068 (6)
O4	0.0508 (8)	0.0346 (6)	0.0330 (6)	−0.0081 (5)	0.0012 (5)	−0.0020 (5)
O5	0.0613 (9)	0.0318 (6)	0.0423 (7)	−0.0088 (6)	−0.0003 (6)	−0.0056 (5)
Br1	0.06364 (16)	0.06784 (16)	0.03773 (12)	−0.02209 (11)	0.00927 (9)	−0.01359 (9)

### Geometric parameters (Å, º)

**Table:**

C1—C6	1.375 (3)	C11—C12	1.444 (2)
C1—C2	1.386 (3)	C12—O5	1.214 (2)
C1—H1	0.9300	C12—O4	1.338 (2)
C2—C3	1.368 (3)	C13—O4	1.442 (2)
C2—H2	0.9300	C13—H13A	0.9600
C3—C4	1.396 (3)	C13—H13B	0.9600
C3—H3	0.9300	C13—H13C	0.9600
C4—C5	1.372 (3)	C14—C19	1.381 (2)
C4—H4	0.9300	C14—C15	1.389 (3)
C5—C6	1.383 (2)	C14—N2	1.405 (2)
C5—C8	1.501 (3)	C15—C16	1.370 (3)
C6—N1	1.409 (2)	C15—H15	0.9300
C7—O1	1.213 (2)	C16—C17	1.366 (3)
C7—N1	1.336 (2)	C16—H16	0.9300
C7—C8	1.563 (2)	C17—C18	1.375 (3)
C8—O2	1.446 (2)	C17—Br1	1.8962 (19)
C8—C11	1.496 (2)	C18—C19	1.379 (3)
C9—O3	1.185 (2)	C18—H18	0.9300
C9—O2	1.357 (2)	C19—H19	0.9300
C9—C10	1.497 (2)	N1—H1A	0.92 (2)
C10—N2	1.343 (2)	N2—H2A	0.84 (3)
C10—C11	1.356 (2)		
			
C6—C1—C2	116.7 (2)	O5—C12—O4	123.21 (16)
C6—C1—H1	121.7	O5—C12—C11	123.72 (16)
C2—C1—H1	121.7	O4—C12—C11	113.02 (14)
C3—C2—C1	122.0 (2)	O4—C13—H13A	109.5
C3—C2—H2	119.0	O4—C13—H13B	109.5
C1—C2—H2	119.0	H13A—C13—H13B	109.5
C2—C3—C4	120.7 (2)	O4—C13—H13C	109.5
C2—C3—H3	119.6	H13A—C13—H13C	109.5
C4—C3—H3	119.6	H13B—C13—H13C	109.5
C5—C4—C3	117.7 (2)	C19—C14—C15	119.37 (17)
C5—C4—H4	121.1	C19—C14—N2	124.10 (16)
C3—C4—H4	121.1	C15—C14—N2	116.49 (16)
C4—C5—C6	120.74 (18)	C16—C15—C14	120.46 (17)
C4—C5—C8	130.56 (18)	C16—C15—H15	119.8
C6—C5—C8	108.70 (14)	C14—C15—H15	119.8
C1—C6—C5	122.12 (17)	C17—C16—C15	119.54 (17)
C1—C6—N1	128.20 (18)	C17—C16—H16	120.2
C5—C6—N1	109.67 (15)	C15—C16—H16	120.2
O1—C7—N1	128.24 (15)	C16—C17—C18	121.04 (18)
O1—C7—C8	124.32 (15)	C16—C17—Br1	118.82 (14)
N1—C7—C8	107.43 (15)	C18—C17—Br1	120.13 (14)
O2—C8—C11	103.78 (14)	C17—C18—C19	119.60 (16)
O2—C8—C5	110.91 (13)	C17—C18—H18	120.2
C11—C8—C5	118.36 (14)	C19—C18—H18	120.2
O2—C8—C7	106.91 (13)	C18—C19—C14	119.96 (17)
C11—C8—C7	114.63 (13)	C18—C19—H19	120.0
C5—C8—C7	101.94 (14)	C14—C19—H19	120.0
O3—C9—O2	121.60 (16)	C7—N1—C6	112.18 (15)
O3—C9—C10	130.94 (17)	C7—N1—H1A	123.8 (14)
O2—C9—C10	107.37 (15)	C6—N1—H1A	123.9 (14)
N2—C10—C11	125.71 (16)	C10—N2—C14	132.62 (16)
N2—C10—C9	126.41 (17)	C10—N2—H2A	107.5 (17)
C11—C10—C9	107.60 (14)	C14—N2—H2A	119.9 (17)
C10—C11—C12	123.83 (15)	C9—O2—C8	111.32 (13)
C10—C11—C8	109.57 (14)	C12—O4—C13	114.86 (14)
C12—C11—C8	126.35 (15)		
			
C6—C1—C2—C3	−0.2 (3)	C7—C8—C11—C10	−120.97 (16)
C1—C2—C3—C4	−0.3 (3)	O2—C8—C11—C12	169.66 (15)
C2—C3—C4—C5	0.6 (3)	C5—C8—C11—C12	−67.0 (2)
C3—C4—C5—C6	−0.4 (3)	C7—C8—C11—C12	53.4 (2)
C3—C4—C5—C8	179.71 (16)	C10—C11—C12—O5	−1.3 (3)
C2—C1—C6—C5	0.4 (3)	C8—C11—C12—O5	−174.99 (16)
C2—C1—C6—N1	−178.36 (18)	C10—C11—C12—O4	176.09 (15)
C4—C5—C6—C1	−0.1 (3)	C8—C11—C12—O4	2.4 (2)
C8—C5—C6—C1	179.81 (16)	C19—C14—C15—C16	1.3 (3)
C4—C5—C6—N1	178.90 (16)	N2—C14—C15—C16	179.03 (17)
C8—C5—C6—N1	−1.22 (19)	C14—C15—C16—C17	0.3 (3)
C4—C5—C8—O2	68.7 (2)	C15—C16—C17—C18	−1.4 (3)
C6—C5—C8—O2	−111.21 (15)	C15—C16—C17—Br1	177.77 (14)
C4—C5—C8—C11	−51.1 (3)	C16—C17—C18—C19	0.7 (3)
C6—C5—C8—C11	129.05 (16)	Br1—C17—C18—C19	−178.39 (13)
C4—C5—C8—C7	−177.83 (18)	C17—C18—C19—C14	0.9 (3)
C6—C5—C8—C7	2.31 (17)	C15—C14—C19—C18	−1.9 (3)
O1—C7—C8—O2	−65.7 (2)	N2—C14—C19—C18	−179.48 (17)
N1—C7—C8—O2	113.77 (15)	O1—C7—N1—C6	−178.44 (17)
O1—C7—C8—C11	48.8 (2)	C8—C7—N1—C6	2.16 (19)
N1—C7—C8—C11	−131.82 (16)	C1—C6—N1—C7	178.22 (18)
O1—C7—C8—C5	177.89 (16)	C5—C6—N1—C7	−0.7 (2)
N1—C7—C8—C5	−2.69 (17)	C11—C10—N2—C14	176.41 (18)
O3—C9—C10—N2	−2.8 (3)	C9—C10—N2—C14	−10.5 (3)
O2—C9—C10—N2	−179.46 (16)	C19—C14—N2—C10	−33.1 (3)
O3—C9—C10—C11	171.27 (19)	C15—C14—N2—C10	149.3 (2)
O2—C9—C10—C11	−5.36 (18)	O3—C9—O2—C8	−174.71 (15)
N2—C10—C11—C12	5.7 (3)	C10—C9—O2—C8	2.31 (17)
C9—C10—C11—C12	−168.42 (15)	C11—C8—O2—C9	1.26 (17)
N2—C10—C11—C8	−179.68 (17)	C5—C8—O2—C9	−126.86 (14)
C9—C10—C11—C8	6.16 (18)	C7—C8—O2—C9	122.79 (14)
O2—C8—C11—C10	−4.75 (17)	O5—C12—O4—C13	−1.4 (2)
C5—C8—C11—C10	118.62 (17)	C11—C12—O4—C13	−178.89 (16)

### Hydrogen-bond geometry (Å, º)

**Table:**

*D*—H···*A*	*D*—H	H···*A*	*D*···*A*	*D*—H···*A*
N2—H2*A*···O5	0.84 (2)	2.06 (3)	2.773 (3)	143 (2)
N1—H1*A*···O1^i^	0.92 (2)	1.96 (2)	2.869 (3)	168 (2)

Symmetry code: (i) −*x*, −*y*+1, −*z*+2.
